# Genotoxicity assessment data for exfoliated buccal cells exposed to mobile phone radiation

**DOI:** 10.1016/j.dib.2017.09.048

**Published:** 2017-09-22

**Authors:** F.M. de Oliveira, A.M. Carmona, C. Ladeira

**Affiliations:** Institute of Cancer Research, School of Tourism and Maritime Technology, Lisbon School of Health Technology, United Kingdom

**Keywords:** Electromagnetic fields, Mobile phones, Genotoxicity, Micronuclei, Exfoliated buccal cells, Feulgen stain

## Abstract

Healthy mobile phone users aged 18–30 y.o. provided exfoliated buccal cells samples from the right and left inner cheeks. A total of 2000 cells per subject were screened for the presence of micronuclei as a sign of genotoxic damage, according to the mobile phone use profile of each user.

**Specifications Table**

**Table:** 

Subject area	*Physics, Biology*
More specific subject area	*Radiation genotoxicity*
Type of data	*Text file, graphs*
How data was acquired	*Leica DM500 Microscope, survey*
Data format	*Analyzed*
Experimental factors	*Histochemical stain with Feulgen's method*
Experimental features	*Microscope screening at 1000x with immersion oil*
Data source location	*Lisbon Metropolitan Area, Portugal, 38.7223* ° N, 9.1393 ° W
Data accessibility	*Data are within this article.*
Related research article	*Genotoxicity assessment of mobile phone radiation in exfoliated buccal cells (in press)*

**Value of the data**

• Data was collected from members of an important share of mobile phone users (young adults, aged 18–30). Establishing the effects of mobile phone use in this population can contribute to an overall perception of how such devices affect the majority of its users [1].
• Improved perception of the effects of mobile phone electromagnetic radiation in humans can contribute to improved safety guidelines for the use of this device and help combat long standing misconceptions on mobile phone radiation [2], [3], [4], [5].
• Establishing the relevance and efficacy of exposure levels and of the biomarker assessment method herein described can help in the determination of a genotoxicity-based model of observation and thus promote the development of new methods.

## 1 Data

**Fig. 1:**
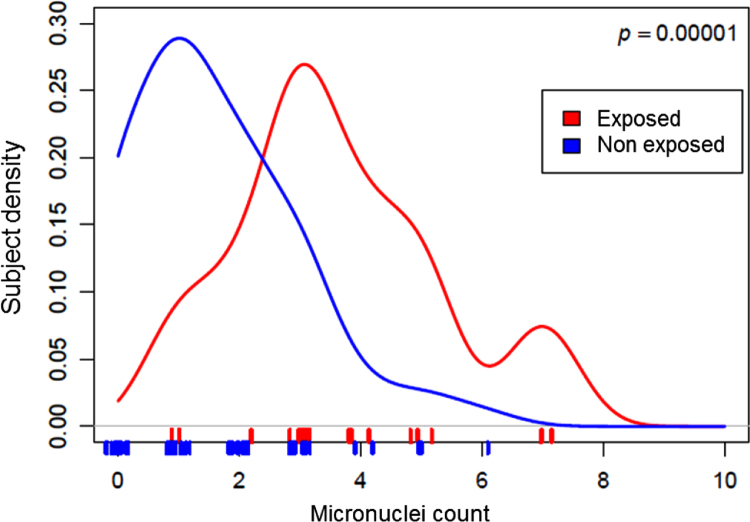
Density plot of micronuclei frequency in subjects exposed and non-exposed to known genotoxic agents.

**Fig. 2:**
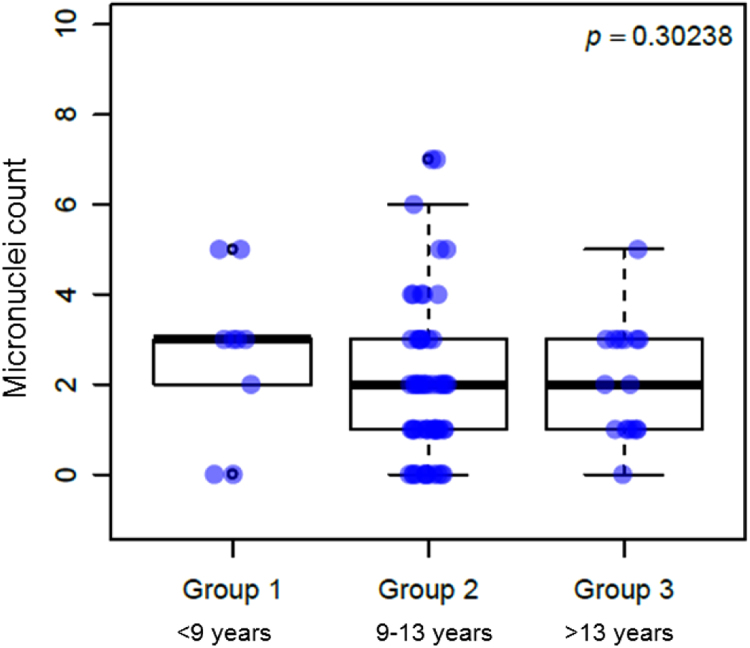
Micronuclei frequency distribution by history of mobile phone use in years.

Overall micronucleus frequency in the study population (2.02 (±1.65) per 2000 cells) was found to be within currently accepted physiological ranges [6]. Lifestyle factors assessed in subjects were not shown to affect the frequency of this genotoxicity biomarker, with the exception of occupational exposure to known genotoxic agents (Fig. 1). Daily duration, side of use and history of mobile phone in years (Fig. 2) did not correlate to higher micronucleus frequencies.

## 2 Experimental design, materials and methods

Buccal exfoliated cells were collected using sterile endobrushes followed by a smearing technique on histological slides. Cells were fixated with an ethanol-based solution, air-dried and stained according to Feulgen's method [7]. Mounted slides were screened by a singleobserver at a 1000x magnification with immersion oil and morphological objects within accepted intervals for micronuclei were counted in the first valid 2000 cells observed [8], [9], [10]. A spreadsheet containing subject characteristics and micronuclei frequencies was used as database for statistical analysis using the Wilcoxon and the Kruskal–Wallis non-parametric tests [11], [12], [13].
