# Supplementary material for: Genotoxicity assessment data for exfoliated buccal cells exposed to mobile phone radiation
Source: Data Brief. 2017 Sep 22;15:344–7. doi: 10.1016/j.dib.2017.09.048 (PMC5712057; doi:10.1016/j.dib.2017.09.048)
Supplement: Supplementary file 1 — Supplementary material [file mmc1.zip › COI_MUTGEN_CL.pdf]

ELSEVIER

Mutation Research-Genetic Toxicology and Environmental Mutagenesis

Conflict of Interest Policy

Manuscript number (if applicable):

Article Title: Genotoxicity assessment of mobile phone radiation in exfoliated buccal cells

Author name: Carina Ladeira

Declarations

Mutation Research-Genetic Toxicology and Environmental Mutagenesis requires that all authors sign a declaration of conflicting interests. If you have nothing to declare in any of these categories then this should be stated.

Conflict of Interest
A conflicting interest exists when professional judgement concerning a primary interest (such as patient's welfare or the validity of research) may be influenced by a secondary interest (such as financial gain or personal rivalry). It may arise for the authors when they have financial interest that may influence their interpretation of their results or those of others. Examples of potential conflicts of interest include employment, consultancies, stock ownership, honoraria, paid expert testimony, patent applications/registrations, and grants or other funding.

Please state any competing interests

The author declare that there are no conflicts of interests.

Funding Source
All sources of funding should also be acknowledged and you should declare any involvement of study sponsors in the study design; collection, analysis and interpretation of data; the writing of the manuscript; the decision to submit the manuscript for publication. If the study sponsors had no such involvement, this should be stated.

Please state any sources of funding for your research

The author declare that there are no sponsoring involvement.

Signature (a scanned signature is acceptable, but each author must sign)

____

Print name

Carina Ladeira
